# Semantic Clinical Artificial Intelligence vs Native Large Language Model Performance on the USMLE

**DOI:** 10.1001/jamanetworkopen.2025.6359

**Published:** 2025-04-22

**Authors:** Peter L. Elkin, Guresh Mehta, Frank LeHouillier, Melissa Resnick, Sarah Mullin, Crystal Tomlin, Skyler Resendez, Jiaxing Liu, Jonathan R. Nebeker, Steven H. Brown

**Affiliations:** 1 Department of Biomedical Informatics, Jacobs School of Medicine and Biomedical Sciences, University at Buffalo, Buffalo, New York; 2 Clinical Informatics, Department of Veterans Affairs, Washington, DC; 3 Roswell Park Comprehensive Cancer Center, Buffalo, New York; 4 Department of Medicine, University of Utah, Salt Lake City; 5 Department of Biomedical Informatics, Vanderbilt University Medical Center, Nashville, Tennessee

## Abstract

**Importance:**

Large language models (LLMs) are being implemented in health care. Enhanced accuracy and methods to maintain accuracy over time are needed to maximize LLM benefits.

**Objective:**

To evaluate whether LLM performance on the US Medical Licensing Examination (USMLE) can be improved by including formally represented semantic clinical knowledge.

**Design, Setting, and Participants:**

This comparative effectiveness research study was conducted between June 2024 and February 2025 at the Department of Biomedical Informatics, Jacobs School of Medicine and Biomedical Sciences, University at Buffalo, Buffalo, New York, using sample questions from the USMLE Steps 1, 2, and 3.

**Intervention:**

Semantic clinical artificial intelligence (SCAI) was developed to insert formally represented semantic clinical knowledge into LLMs using retrieval augmented generation (RAG).

**Main Outcomes and Measures:**

The SCAI method was evaluated by comparing the performance of 3 Llama LLMs (13B, 70B, and 405B; Meta) with and without SCAI RAG on text-based questions from the USMLE Steps 1, 2, and 3. LLM accuracy for answering questions was determined by comparing the LLM output with the USMLE answer key.

**Results:**

The LLMs were tested on 87 questions in the USMLE Step 1, 103 in Step 2, and 123 in Step 3. The 13B LLM enhanced by SCAI RAG was associated with significantly improved performance on Steps 1 and 3 but only met the 60% passing threshold on Step 3 (74 questions correct [60.2%]). The 70B and 405B LLMs passed all the USMLE steps with and without SCAI RAG. The SCAI RAG 70B model scored 80 questions (92.0%) correctly on Step 1, 82 (79.6%) on Step 2, and 112 (91.1%) on Step 3. The SCAI RAG 405B model scored 79 (90.8%) correctly on Step 1, 87 (84.5%) on Step 2, and 117 (95.1%) on Step 3. Significant improvements associated with SCAI RAG were found for the 13B model on Steps 1 and 3, the 70B model on Step 2, and the 405B parameter model on Step 3. The 70B model was significantly better than the 13B model, and the 405B model was not significantly better than the 70B model.

**Conclusions and Relevance:**

In this comparative effectiveness research study, SCAI RAG was associated with significantly improved scores on the USMLE Steps 1, 2, and 3. The 13B model passed Step 3 with RAG, and the 70B and 405B models passed and scored well on Steps 1, 2, and 3 with or without augmentation. New forms of reasoning by LLMs, like semantic reasoning, have potential to improve the accuracy of LLM performance on important medical questions. Improving LLM performance in health care with targeted, up-to-date clinical knowledge is an important step in LLM implementation and acceptance.

## Key Points

**Question:**

Can large language model (LLM) performance on the US Medical Licensing Examination (USMLE) be improved by including formally represented semantic clinical knowledge?

**Findings:**

In this comparative effectiveness research study of 3 LLMs, semantic clinical knowledge was associated with improved LLM performance on questions on the USMLE Steps 1, 2, and 3. Scores on 2 of the models exceeded 60% correctness with or without semantic augmentation.

**Meaning:**

The findings suggest that future health care education and delivery will likely be impacted by artificial intelligence that works in partnership with clinicians.

## Teaser

This comparative effectiveness research study evaluates how large language models enhanced by semantic clinical artificial intelligence (AI) perform on the US Medical Licensing Examination (USMLE).

## Introduction

### Large Language Model Overview

Large language models (LLMs) are a specialized type of neural network that excels at transforming input strings (eg, questions) into responsive output strings (answers).^1,2,3^ LLMs are trained to predict the next word in a sentence by analyzing terabytes of text and adjusting billions of internal probabilistic parameters to create output responses.

Two fundamental technical advances underpin LLMs. Vector embedding is a way to represent words, phrases, and entire sentences so that a computer can learn the relationships among them.^4^ Each word is processed into a vector of related words and weights. Vectors help the LLM mathematically find correlations between words in context. Attention mechanisms are a second fundamental advance.^5^ LLMs use attention mechanisms to focus on the most important words in a sentence when making predictions. Instead of treating every word equally, attention mechanisms assign different levels of importance to each word based on its relevance to the current context.

Methods to improve LLM performance include increased model size (number of parameters), dataset size, and amount of computational power used for training.^2,3,6^ Prompt engineering, tuning, and prompt distillation methods have been shown to impact LLM performance,^7,8^ as have ensemble methods.^9^ Retrieval-augmented generation (RAG), described by Lewis et al,^10^ is a method of enriching LLMs with pretrained knowledge as part of the input prompt to enhance performance on knowledge-intensive tasks, including the reduction of hallucinations.^10,11,12,13,14^ RAG allows integration of relevant, up-to-date, and domain-specific knowledge (eg, clinical knowledge) into LLMs and avoids expensive foundational model retraining. RAG requires methods to generate and integrate input text within LLM-specific size limits and can increase costs of inferencing.^15^

### LLMs in Answering Health Care Questions

Numerous articles have addressed the ability of LLMs to answer clinical questions from sources including the US Medical Licensing Examination (USMLE),^16,17,18^ USMLE-style questions,^19,20^ neurology board–style examinations,^21^ anesthesia board–style examinations,^22^ the national premedical examination in India,^23^ the Chinese postgraduate medical examination,^24^ orthopedic in-service training,^25^ surgical questions,^26^ and *JAMA* and *The New England Journal of Medicine* clinical vignettes.^27^ One commonly mentioned challenge is the need to enhance LLM accuracy and reliability in question-answer tasks^9^ and differential diagnosis.^28^

### Semantic Triples and Knowledge Graphs for Clinical Knowledge

Clinical domain–specific knowledge, needed for question answering and differential diagnosis, can be represented as knowledge graphs based on formal logic. According to Murali et al,^29^ “a knowledge graph crafted from medical concepts, events, and relationships acts as a medical semantic network to extract new links and hidden patterns from health data sources.” Nodes in the graph represent concepts, such as a medication or disease, and arcs represent relations between concepts, such as *treats* (ie, 1 concept treats another). Well-known medical semantic networks include Systematized Nomenclature of Medicine–Clinical Terms (SNOMED CT),^30^ the Unified Medical Language System,^31^ and DrugBank.^32^

**Figure 1.**
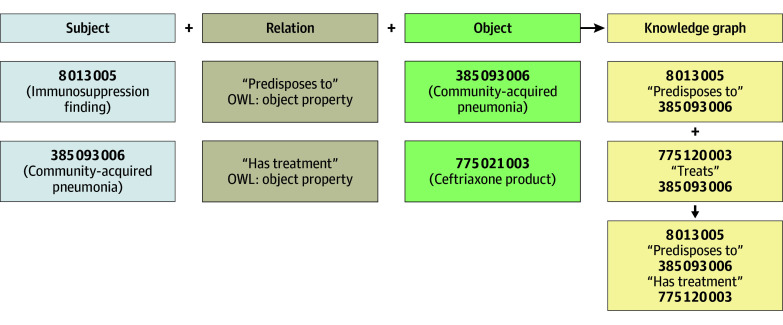
Semantic Triples and Knowledge Graphs The knowledge graphs may contain 1 or more semantic triples. Two semantic triples are combined at the lower right of the figure. Numbers signify Systematized Nomenclature of Medicine–Clinical Terms codes. OWL indicates W3C Web Ontology Language.

Semantic triples are a simple form of a semantic network. Semantic triples take the form subject-relation-object (eg, penicillin [subject] treats [relation] pneumococcal pneumonia [object]) (Figure 1). When semantic networks are represented with formal logic (eg, description logic), it is possible to draw logical inferences (ie, to reason) from them using software tools, such as classifiers.^33^ Classification tasks are part of many types of reasoning.

### Study Objective

We used RAG and semantic triples to develop a method named semantic clinical artificial intelligence (SCAI; pronounced *sky*) to semantically augment transformer-based LLMs. Our hypothesis was that augmenting native LLMs with relevant clinical knowledge as semantic triples would improve accuracy and decrease confabulation. We tested our hypothesis between June 2024 and February 2025 by comparing the performance of 3 native LLMs against their SCAI RAG–enhanced counterparts on Steps 1, 2, and 3 of the USMLE. The USMLE, required for licensure of allopathic physicians in all 50 states, is a 3-step examination taken throughout a medical student’s education. The USMLE “assesses a physician’s ability to apply knowledge, concepts, and principles, and to demonstrate fundamental patient-centered skills, that are important in health and disease and that constitute the basis of safe and effective patient care.”^34^

## Methods

### Overview

**Figure 2.**
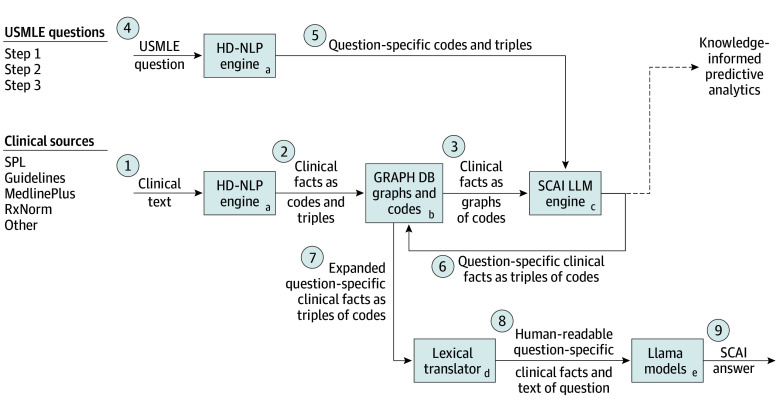
Clinical Knowledge Training and Retrieval Augmented Generation (RAG) Prompt Generation for Semantic Clinical Artificial Intelligence (SCAI) Implementation Numbered circles represent data flows between algorithms and/or data stores, which are represented by boxes with lowercase letters. GRAPH DB indicates graph database; HD-NLP, high-definition natural language processing; LLM, large language model; SPL, structured product labeling; USMLE, United States Medical Licensing Examination.

In this comparative effectiveness research study conducted in the Department of Biomedical Informatics at the Jacobs School of Medicine and Biomedical Sciences, University at Buffalo, Buffalo, New York, SCAI was developed by using high-definition natural language processing (HD-NLP) to parse clinical references and USMLE practice questions.^35,36^ We processed the resulting codes and semantic triples into an embedding of graphs and knowledge graphs and used them to generate English-language sentences for each USMLE question containing question-specific clinical knowledge for presentation to the LLM. Our RAG technique uses formal semantics to inform LLM processing. This study was deemed exempt by the University at Buffalo institutional review board because no patient data were used. We followed the International Society for Pharmacoeconomics and Outcomes Research (ISPOR) reporting guideline for comparative effectiveness studies.^37^ Detailed descriptions for each step follow. The study workflow (Figure 2) shows both the knowledge generation process (steps 1-3) and the workflow for LLM prompting (steps 4-8).

### USMLE Data Collection and Curation

We used USMLE sample questions to create our study’s input text files.^38,39,40^ Questions were excluded from the dataset if they possessed some type of visual component (Table 1).

**Table 1.** Curation of USMLE Questions, Including Counts and Reasons for Question Exclusion

USMLE step	Date updated	Questions, No.	Exclusion reason (No. [%])		Used in study, No. (%)
			Visual	Nonvisual	
Step 1	June 2022	119	Total (29 [24.4]): photograph (8 [27.6]), graph (5 [17.2]), photomicrograph (4 [13.8]), multipart answer with directional arrows (3 [10.3]), radiograph (2 [6.9]), diagram (2 [6.9]), CT scan (1 [3.4]), MRI (1 [3.4]), Doppler image (1 [3.4]), bar graph (1 [3.4]), and ultrasonography (1 [3.4])	Total (3 [2.5]): multipart answer not conducive to a text file format	87 (73.1)
Step 2	July 2023	120	Total (14 [11.7]): photograph (6 [42.9]), radiograph (4 [28.6]), cardiac rhythm strip (1 [7.1]), fetal heart tracing (1 [7.1]), hysterosalpingography (1 [7.1]), and stool smear (1 [7.1])	Total (3 [2.5]): complex table not conducive to a text file format	103 (85.8)
Step 3	August 2022	137	Total (11 [8.0]): radiograph (5 [45.5]), photograph (4 [36.4]), family pedigree (1 [9.1]), and ECG component (1 [9.1])	Total (3 [2.2]): complex 2-page pharmaceutical advertisement (2 [66.7]) and complex table not conducive to a text file format (1 [33.3])	123 (89.8)

Abbreviations: CT, computed tomography; EGC, electrocardiography; MRI, magnetic resonance imaging; USMLE, US Medical Licensing Examination.

### HD-NLP System

We used HD-NLP to assign ontology terms to text in clinical references and USMLE questions (Figure 2). HD-NLP extracts information from natural language text and stores the information using off-the-shelf biomedical ontologies, including Basic Formal Ontology^41^ as an upper-level ontology, Ontology for Biomedical Investigations,^42^ SNOMED CT,^30^ LOINC,^43^ RxNorm,^44^ and their superset, Solor.^35^

HD-NLP uses syntactic processing to match text with ontologic terms and language models for linguistic representation (eFigure 1 in Supplement 1). We used an English-language model to identify sentences, phrases, words, and parts of speech. Terms from the input ontologies were assigned to spans of text. String matching techniques allowed for inexact matches influenced by the underlying language model (eFigure 3 in Supplement 1). HD-NLP then built graphs and knowledge graphs from the generated codes and semantic triples (Figure 2). HD-NLP has a published sensitivity (recall) of 99.7% and a positive predictive value (precision) of 99.8% in translating clinical problems from electronic health records into codified data.^45^

We used HD-NLP to create USMLE question–specific codes and semantic triples for use in generating question-specific RAG content (Figure 2). We then used HD-NLP to create clinical knowledge codes and semantic triples from Agency for Healthcare Research and Quality and ECRI clinical guidelines,^46^ MedlinePlus content hosted by the National Library of Medicine (NLM),^47^ Structured Product Labeling from the NLM DailyMed database,^48^ order sets,^49^ checklists,^50^ the biomedical literature (SemMedDB),^51,52^ and the Comparative Toxicogenomics Database^53^ (Figure 2).

### Embedding Knowledge Graphs

We developed a corpus of clinical knowledge embeddings by training on SNOMED CT, RxNorm, and the clinical knowledge codes and semantic triples created by HD-NLP (Figure 2 and eFigure 2 in Supplement 1) via the application of multiple complementary algorithms (ie, ensemble processing). We trained embeddings using 2 methods: graph embeddings (for pairs of entities with no differentiation on relations) and knowledge graph embeddings (for subject-relation-object triples). We used a modified node2vec,^54^ which we named Sno2Vec,^55^ and DeepWalk^56^ graph embedding algorithms to create subject-relation and object-relation embeddings from deconstructed triples. We used the same graph algorithms to embed pairs of codes co-occurring in the same sentence in the input text. We used TransE^57^ and RotatE^58^ as knowledge graph–embedding algorithms to create subject-relation-object embeddings. We used the GraphVite^59^ implementation to train our knowledge graph embeddings. For all embedding models, we varied the embedded dimensions as *d* = (128, 256, 512), although embeddings with 512 dimensions were superior in all tasks. These embeddings were then used to generate associated codes and knowledge from the codes identified in the input question for the SCAI pipeline (Figure 2). We called the results of this training the SCAI LLM semantic knowledge reasoner (eFigure 4 in Supplement 1).

### Semantic Data Processing for RAG

We created human-readable USMLE question-specific clinical knowledge for RAG via the following steps (Figure 2). We processed each USMLE question using the HD-NLP system to extract relevant medical codes. We used the resulting codes to retrieve associated embeddings from our corpus of embeddings, using cosine similarity search to identify the most similar medical codes. We presented the resulting codes to the graph database to retrieve additional related semantic triples. We filtered out a subset of triples based on their relationship types (eg, IsA and “part of” relationships) to reduce final RAG file size while preserving high-value content (Table 2). We then translated the retrieved triples into a human-readable format for presentation to the LLM model (Figure 2).

**Table 2.** Relations and Their Frequencies in the Embedded Semantic Triples

Relation	Embedded triples, No.	RAG use
Total	13 687 849	5 349 778
Causes	75 927	Yes
Clinical course of	7860	Yes
Definitional manifestation of	59 927	Yes
Diagnoses	29 128	Yes
Finding method of	35 077	Yes
Has adverse reaction	4 063 753	Yes
Has contraindication	925 078	Yes
Has indication	36 778	Yes
Occurs in	39 128	Yes
Pathological process of	46 896	Yes
Predisposes	15 248	Yes
Prevents	4846	Yes
Treats	57 028	Yes
Approach of	4638	No
Associated etiologic finding of	3258	No
Associated finding of	26 973	No
Associated with	32 384	No
Characterizes	266	No
Component of	4318	No
Direct morphology of	48 988	No
Direct procedure site of	95 600	No
Entire anatomy structure of	29 212	No
Finding site of	443 856	No
Focus of	32 154	No
Has part	120 694	No
Indirect morphology of	3028	No
Indirect procedure site of	31 018	No
Inheres in	386	No
Intent of	45 738	No
Interprets	200 184	No
IsA	4 826 679	No
Method of	620 518	No
Occurs after	32 498	No
Occurs before	32 498	No
Part of	4634	No
Procedure morphology of	2152	No
Procedure site of	156 708	No
Surgical approach of	1440	No
Temporally followed by	6558	No
Temporally follows	13 686	No

Abbreviation: RAG, retrieval augmented generation.

### LLM Evaluation

We tested the Meta Llama 2 13B parameter model,^60,61^ the Meta Llama 3 70B parameter model,^62^ and the Meta Llama 3.1 405B parameter model^63^ on USMLE Steps 1, 2, and 3 with and without the SCAI RAG. We presented each USMLE question to each native LLM (the aforementioned models) and recorded the model’s answer and its correctness. We repeated this process with the addition of USMLE question-specific clinical knowledge for SCAI RAG.

### Statistical Analysis

Treatment cohorts were defined and classified (RAG vs not). Scoring from a gold standard answer key was automated with secondary human review (P.L.E., G.M., and F.L.). We defined confabulation as answering a question (vs remaining silent) with the wrong answer (ie, a false-positive response). We measured the confabulation rate as the count of wrong nonnull answers. We used the exact Cochran *Q* test with blocks for questions given that the difficulty of questions varies across questions and questions may be clustered to determine the significance of the differences in test scores and confabulation rates. Analyses were done using ExamEvaluator, version 1 (in-house software). Two-sided *P* < .05 was considered significant.

## Results

### Embeddings

Table 2 summarizes the clinical knowledge semantic triples based on the type of relation linking subject and object. Overall, we created and embedded 13 687 849 semantic triples with 40 different relations. To constrain RAG text size, we limited triple generation to the 13 relationship types (defining 5 349 778 embeddings) that we felt most appropriate for answering USMLE questions (Figure 2).

### LLM Performance on USMLE Steps

In the USMLE Steps 1, 2, and 3, there were 87, 103, and 123 text-based questions, respectively. The native 13B parameter LLM failed to meet the passing grade of 60% for any examination (Table 3). The SCAI RAG–enhanced 13B LLM showed substantially improved performance on the USMLE Steps 1 and 3 but only exceeded the 60% passing threshold on Step 3 (74 of 123 questions [60.2%]).

**Table 3.** Performance and Confabulation Rate of Each Large Language Model With and Without SCAI RAG on USMLE

USMLE performance	13B model				70B model				405B model			
	Questions answered, No. (%)		Cochran *Q*	*P* value	Questions answered, No. (%)		Cochran *Q*	*P* value	Questions answered, No. (%)		Cochran *Q*	*P* value
	Native model	With SCAI RAG			Native model	With SCAI RAG			Native model	With SCAI RAG		
Step 1 (87 questions)												
Correct	29 (33.3)	48 (55.2)	15.70	.001	75 (86.2)	80 (92.0)	6.08	.02	77 (88.5)	79 (90.8)	0.50	.71
Confabulation	58 (66.7)	39 (44.8)			12 (13.8)	7 (8.0)			10 (11.5)	8 (9.2)		
Step 2 (103 questions)												
Correct	50 (48.5)	56 (54.4)	1.29	.36	76 (73.8)	82 (79.6)	4.50	.04	81 (78.6)	87 (84.5)	2.25	.22
Confabulation	53 (51.5)	47 (45.6)			27 (26.2)	21 (20.4)			22 (21.4)	16 (15.5)		
Step 3 (123 questions)												
Correct	59 (48.0)	74 (60.2)	6.08	.02	107 (87.0)	112 (91.1)	3.57	.11	107 (87.0)	117 (95.1)	8.33	.006
Confabulation	64 (52.0)	49 (39.8)			16 (13.0)	11 (8.9)			16 (13.0)	6 (4.9)		

Abbreviations: RAG, retrieval augmented generation; SCAI, semantic clinical artificial intelligence; USMLE, US Medical Licensing Examination.

The 70B parameter and 405B parameter models passed each USMLE step with or without SCAI RAG. The SCAI RAG 70B model scored 80 of 87 questions (92.0%) correctly on Step 1, 82 of 103 (79.6%) on Step 2 (USMLE test score, 261), and 112 of 123 (91.1%) on Step 3 (USMLE test score, 253).^64^ The SCAI RAG 405B model scored 79 of 87 questions (90.8%) correctly on Step 1, 87 of 103 (84.5%) on Step 2 (USMLE test score, 264), and 117 of 123 (95.1%) on Step 3 (USMLE test score, 258).^64^ Significant improvements with SCAI RAG were found for the 70B parameter model on Steps 1 and 2. The 70B model performed significantly better than the 13B model (Step 1: 48 of 87 questions correct [55.2%]; Step 2: 56 of 103 [54.4%]; 74 of 123 [60.2%]; *P* < .001 for all) and the 405B model was not significantly better than the 70B model (Step 1: 79 or 87 questions correct [90.8%] [*P* = .64]; Step 2: 97 of 103 [84.5%] [*P* = .24]; Step 3: 117 of 123 [95.1%] [*P* > .99]). The SCAI confabulation (error) rate was as low as 6 of 123 questions (4.9%) for the 405B model on Step 3.

### SCAI RAG Example

#### USMLE Input Prompt

The following is a sample input prompt for the USMLE^40^ (a second example is given in the eAppendix in Supplement 1):

A 24-year-old recent college graduate comes to the office because of a 3-year history of increased frequency of urination and occasional incontinence whenever she travels. She says the urinary symptoms typically begin a few days before she is going to fly and they stop the day after she arrives at her destination. She says she is anxious about flying. She reports having had similar but milder symptoms prior to examinations before graduating. Medical history is otherwise unremarkable. Her only medication is an oral contraceptive. Vital signs are normal. Physical examination discloses no abnormalities. Urinalysis and urine culture are normal. Which of the following is the most appropriate next step? (A) Recommend behavioral therapy, (B) Recommend psychoanalytic psychotherapy, (C) Recommend that the patient avoid any stressful activities that cause the problem, (D) Review the patient’s sexual history, or (E) Reassure the patient that her symptoms will resolve in time.

#### Native 13B Incorrect Answer

The native 13B model provided the following incorrect answer and justification:

Based on the information provided, the most appropriate next step would be: (E) Reassure the patient that her symptoms will resolve in time. The patient’s symptoms of increased frequency of urination and occasional incontinence are likely related to her anxiety about flying, as they occur only before and during travel. The fact that the symptoms resolve after the patient arrives at her destination suggests that they are related to stress and anxiety rather than a physical condition. Recommending behavioral therapy (A) or psychoanalytic psychotherapy (B) may be appropriate in the long term, but it is not the most appropriate next step at this time. Recommending that the patient avoid stressful activities (C) may also be a viable option, but it does not address the underlying cause of the patient’s symptoms. Reviewing the patient’s sexual history (D) is not relevant to the patient’s symptoms and would not be an appropriate next step. Therefore, the best option is to reassure the patient that her symptoms will resolve in time and provide her with support and resources to manage her anxiety.

#### SCAI Clinical Context for RAG

The SCAI clinical context for RAG was as follows: *“Abnormal vital signs” (finding) predisposes one to “pneumonia” (disorder), “urinalysis” (procedure) diagnoses “crystalluria” (finding), “urinalysis” (procedure) diagnoses “nephrotic range proteinuria” (finding),* and *“uses OC (oral contraceptive)” causes “amenorrhea” (finding).* For the full file, see the eAppendix in Supplement 1.

#### 13B With SCAI LLM Correct Answer

The 13B model with SCAI LLM provided the following correct answer and justification:

Based on the patient’s symptoms and the fact that urinalysis and urine culture are normal, the most appropriate next step would be to (A) Recommend behavioral therapy. The patient’s symptoms of urinary frequency and urgency, as well as her history of similar symptoms in the past, suggest that she may be experiencing stress-related urinary dysfunction. Behavioral therapy, such as bladder training or stress management techniques, may be helpful in addressing these issues. While (B) Recommend psychoanalytic psychotherapy may also be appropriate, it is not specifically focused on addressing the patient’s urinary symptoms and may not be the most direct course of action. (C) Recommend that the patient avoid any stressful activities that cause the problem may be a reasonable suggestion, but it does not address the underlying cause of the patient’s symptoms. (D) Review the patient’s sexual history is not directly related to the patient’s symptoms and is not the most appropriate next step. (E) Reassure the patient that her symptoms will resolve in time may be appropriate, but it does not address the underlying cause of the patient’s symptoms or provide a specific course of action.

## Discussion

We developed and evaluated a method for inserting semantic triple–based reasoning into LLMs using RAG. We used formal semantics based on a description logic to inform the LLM with relevant clinical knowledge needed to understand and respond to the question being asked. We compared this against submitting USMLE text-based questions alone to determine whether adding semantics to 3 different LLMs was associated with improved performance on USMLE Steps 1, 2, and 3. We found that our SCAI RAG implementation was associated with statistically significantly better performance than the native LLM (without the contextual semantic information; eg, the addition of semantics as prompt engineering was uniformly associated with improvement, and in many cases, that improvement was statistically significant).

The 13B model was only able to achieve the accuracy threshold of 60% needed to pass USMLE Step 3 with SCAI RAG. Despite this, significant increases in performance on Steps 1 and 3 were associated with using formally represented clinical knowledge via SCAI RAG.

The 70B and 405B models generated scores in the range needed by individuals applying to competitive residency programs even without SCAI RAG. Although the larger base models did well, improved performance was associated with adding the SCAI semantics as RAG. The SCAI confabulation (error) rate was as low as 4.9%. In comparison, Shojania et al^65^ found a 23.5% median error rate of important clinically missed diagnoses at autopsy.

Three studies evaluated ChatGTP 3.5 (OpenAI) (170B parameters) on USMLE text-based questions.^16,17,18^ Kung et al^16^ found scores for Steps 1, 2CK, and 3 to be 45.4%, 54.1%, and 61.5%, respectively. Yaneva et al^17^ found average scores for Steps 1, 2CK, and 3 to be 68.1%, 70.1%, and 61.8%, respectively. Gilson et al^18^ found scores for Steps 1 and 2CK to be 64.4% and 57.8%, respectively. In studies of USMLE-style questions, Bicknell et al^19^ found GPT-4o (200B parameters) to score 90.4%, outperforming the GPT-4 (175B parameters) score of 81.1% and GPT-3.5 score of 60.0%. Brin et al^20^ found that GPT-4 outperformed ChatGPT 3.5 (90% vs 62.5%). Although larger LLMs perform better, there is a cost-benefit tradeoff given their greater hardware and energy requirements. Using RAG to provide updated, domain-specific knowledge may also permit large foundational models to produce correct answers without costly retraining.

The rapid evolution of LLMs and their impressive, better-than-many-humans performance on difficult tasks raises the following questions: do LLMs reason, and are they intelligent? For many years, the Turing test was considered the gold standard for artificial intelligence to be considered to have a human-level intellect. The Turing test, originally called the imitation game, is passed when an observer who is conversing with artificial intelligence cannot tell whether they are conversing with a human or a machine.^66^ LLMs have passed the Turing text in some evaluations.^67,68^

The idea of semantic networks in clinical reasoning—how expert physicians structure their knowledge in interconnected networks, linking symptoms, diseases, and clinical findings in meaningful ways—has been documented.^69,70^ Bordage and Lemieux^71^ found that “the successful diagnosticians, either students or specialists, are those who use the most diversified sets of abstract relationships and, therefore, who have broader or deeper representations of the problem.” This finding parallels our findings regarding the use of semantic augmentation via RAG to enable semantic reasoning vs a foundational model alone in the present study. Even in the absence of semantic triples, cosine similarity and vector operations provide limited semantic capabilities, such as disambiguation.

LLMs can use chain-of-thought reasoning to solve complex problems that require step-by-step logic in fields such as math, logic, planning, and commonsense reasoning.^72,73,74^ Chain-of-thought reasoning generates human-readable explanations for intermediate steps that improve performance and interpretability of final answers.

We offer 2 arguments in favor of the assertion that semantically augmented LLMs can reason. First, clinical reasoning can be represented mathematically.^75^ Using pretest probability and the sensitivity and specificity of a test, one can calculate the posttest probability of disease given a test result using the positive and negative likelihood ratios. In the LLMs used in this study, the similarity between words is calculated as the cosine similarity between 2 vectors *x,y*: Scos(*x*,*y*) = ⟨*x*,*y*⟩∥*x*∥∥*y*∥.

Cosine similarity is used to limit the retrieval set, and then the vector similarities are compared directly. Cosine similarity is comparable to specificity of one term for others. The matrix multiplication operations across vectors are influenced by the number of words in common, which we argue is comparable to sensitivity. Given the parallels to accepted mathematical expressions used in clinical reasoning for evidence-based medicine, we conjecture that semantically augmented LLMs may also be able to perform evidence-based medicine reasoning.

Our second argument compares LLM vector processing with description logic classification, which is considered a form of reasoning because it enables the derivation of logical conclusions via inferences drawn from explicitly defined concepts and relationships. The semantically augmented LLM uses semantic triples and graphs (using distances between multiple words and the next word) and knowledge graphs (using full semantic triples) to classify and categorize cases. As the vectors in the LLM learn the relationships between subjects, relations, and objects, they incorporate those probabilities into their matrices. In the deep layers of the LLM, these semantic triples will have interactions based on their use and will allow the LLM to operate on the logical relations that they have encountered. We posit that this type of classification can be viewed as reasoning in a similar way as a description logic reasoner classifies an ontology.

The pragmatic perspective is that it really does not matter whether one believes that SCAI LLMs can reason or not, as long as they are useful. Of importance is not only what can be done but what should be done with these models. First, we believe these models will augment but not replace clinicians. However, a clinician who uses artificial intelligence may replace a clinician who does not. It is essential that while these powerful engines continue to improve and have the potential to democratize health information globally, the safety of such use is ensured. We believe that clinical informaticians should be involved in the governance and creation of safeguards for the development of health-related artificial intelligence models.

### Limitations

The major limitation of this study is the use of only 3 LLMs. We would like to extend our work to other LLMs to confirm its generalizability. Our down-selection of triples for RAG inclusion based on relationship types may have yielded different results had we selected differently. Practice-based evaluation of SCAI LLM would yield better evidence regarding its ability to assist practicing clinicians.

## Conclusions

In this comparative effectiveness research study, we found that semantic augmentation using SCAI RAG was associated with significantly improved scores on USMLE Steps 1, 2, and 3. The 70B and 405B models were able to pass and score well on all USMLE steps. The addition of semantics via RAG was uniformly an improvement. Using targeted, up-to-date clinical knowledge to improve LLM performance in health care is an important step in the implementation and acceptance of LLMs.

## Notes

Supplement 1. **eAppendix.** SCAI Example **eFigure 1.** High-Level HD-NLP Architecture **eFigure 2.** Logic Flow for the HD-NLP Pipeline **eFigure 3.** Logic Flow Including HD-NLP and Data Ingestion Using HD-NLP and Structured Data **eFigure 4.** Semantic Clinical Artificial Intelligence Interface With Text or Voice Recognition Input and Answers

Supplement 2.
Data Sharing Statement
