# Supplementary material for: Semantic Clinical Artificial Intelligence vs Native Large Language Model Performance on the USMLE
Source: JAMA Netw Open. 2025 Apr 22;8(4):e256359. doi: 10.1001/jamanetworkopen.2025.6359 (PMC12015668; doi:10.1001/jamanetworkopen.2025.6359)
Supplement: Supplement 1. — eAppendix. SCAI Example eFigure 1. High-Level HD-NLP Architecture eFigure 2. Logic Flow for the HD-NLP Pipeline eFigure 3. Logic Flow Including HD-NLP and Data Ingestion Using HD-NLP and Structured Data eFigure 4. Semantic Clinical Artificial Intelligence Interface With Text or Voice Recognition Input and Answers [file jamanetwopen-e256359-s001.pdf]

Supplementary Online Content

Elkin PL, Mehta G, LeHouillier F, et al. Semantic clinical artificial intelligence vs native large language model performance on the USMLE. *JAMA Netw Open*. 2025;8(4):e256359. doi:10.1001/jamanetworkopen.2025.6359

eAppendix. SCAI Example

eFigure 1. High-Level HD-NLP Architecture

eFigure 2. Logic Flow for the HD-NLP Pipeline

eFigure 3. Logic Flow Including HD-NLP and Data Ingestion Using HD-NLP and Structured Data

eFigure 4. Semantic Clinical Artificial Intelligence Interface With Text or Voice Recognition Input and Answers

This supplementary material has been provided by the authors to give readers additional information about their work.

© 2025 Elkin PL et al. *JAMA Network Open*.

eAppendix. SCAI Example

An example of the difference in output between the native 70B parameter Llama model and the SCAI model can be illustrated with the question below:

You: how long should one treat proteus prostatitis?

Native LLM: **Concise Answer with Brief Explanation** **Treatment Duration for Prostatitis (Prostate Inflammation):** * **Acute Bacterial Prostatitis:** + Antibiotics: assistant I apologize, but it seems like your question was cut off. Here's a rewritten, concise answer with a brief explanation, assuming the question was about the treatment duration for prostatitis (prostate inflammation): **Treatment Duration for Prostatitis (Prostate Inflammation):** * **Acute Bacterial Prostatitis:** + **Antibiotics:** - **Mild cases:** - **Mild cases:** - **Duration:** - **Duration:** - **Duration:** - **Duration:** * **Chronic Prostatitis/Pelvic Pain Syndrome:** + **Multimodal therapy (pain management, lifestyle modifications, physical therapy):** - **Duration:** **Detailed Answer:** * **Acute Bacterial Prostatitis:** + **Antibiotics:** - **Mild cases:** - **Duration:** - **Duration:** * **Chronic Prostatitis/Pelvic Pain Syndrome:** + **Multimodal therapy (pain management, lifestyle modifications, physical therapy):** - **Duration:** **Detailed Answer:** * **Acute Bacterial Prostatitis:** + **Antibiotics:** - **Mild cases:** - **Duration:** * **Chronic Prostatitis/Pelvic Pain Syndrome:** + **Multimodal therapy (pain management, lifestyle modifications, physical therapy):** - **Duration:** **Detailed Answer:** * **Acute Bacterial Prostatitis:** + **Antibiotics:** - **Mild cases:** - **Duration:** * **Chronic Prostatitis/Pelvic Pain Syndrome:** + **Multimodal therapy (pain management, lifestyle modifications, physical therapy):** - **Duration:** **Detailed Answer:** * **Acute Bacterial Prostatitis:** + **Antibiotics:** - **Mild cases:** - **Duration:** * **Chronic Prostatitis/Pelvic Pain Syndrome:** + **Multimodal therapy (pain management, lifestyle modifications, physical therapy):** - **Duration:** **Detailed Answer:** * **Acute Bacterial Prostatitis:** + **Antibiotics:** - **Mild cases:** - **Duration:** * **Chronic Prostatitis/Pelvic Pain Syndrome:** + **Multimodal therapy (pain management, lifestyle modifications, physical therapy):** - **Duration:** **Detailed Answer:** * **Acute Bacterial Prostatitis:** + **Antibiotics:** - **Mild cases:** - **Duration:** * **Chronic Prostatitis/Pelvic Pain Syndrome:** + **Multimodal therapy (pain management, lifestyle modifications, physical therapy):** - **Duration:** **Detailed Answer:** * **Acute Bacterial Prostatitis:** + **Antibiotics:** - **Mild cases:** - **Duration:** * **Chronic Prostatitis/Pelvic Pain Syndrome:** + **Multimodal therapy (pain management, lifestyle modifications, physical therapy):** - **Duration:** **Detailed Answer:** * **Acute Bacterial Prostatitis:** + **Antibiotics:** - **Mild cases:** - **Duration:** * **Chronic Prostatitis/Pelvic Pain Syndrome:** + **Multimodal therapy (pain management, lifestyle modifications, physical therapy):** - **Duration:** **Detailed Answer:** * **Acute Bacterial Prostatitis:** + **Antibiotics:** - **Mild cases:** - **Duration:** * **Chronic Prostatitis/Pelvic Pain Syndrome:** + **Multimodal therapy (pain management, lifestyle modifications, physical therapy):** - **Duration:** **Detailed Answer:** * **Acute Bacterial Prostatitis:** + **Antibiotics:** - **Mild cases:** - **Duration:** * **Chronic Prostatitis/Pelvic Pain Syndrome:** + **Multimodal therapy (pain management, lifestyle modifications, physical therapy):** - **Duration:** **Detailed Answer:** * **Acute Bacterial Prostatitis:** +

You: how long should one treat proteus prostatitis?

SCAI: **Concise Answer with Brief Explanation** **Treatment Duration for Prostatitis (Chronic Prostate Pain):** * **Acute Bacterial Prostatitis:** 4-6 weeks of antibiotics, followed by reassessment. * **Chronic Bacterial Prostatitis:** 12 weeks or more of antibiotics, with periodic evaluations. * **Chronic Non-Bacterial Prostatitis (CPPS):** + **Initial Phase (6-12 weeks):** Multimodal therapy (pain management, alpha-blockers, physical therapy, etc.) + **Maintenance Phase:** Ongoing management (3-6 months or longer), adjusting treatments as needed, with regular follow-ups (every 3-6 months). **Important Note:** * Treatment duration may vary depending on individual response, underlying causes, and presence of complications. * Regular follow-ups with a healthcare provider are crucial to adjust treatment plans and monitor for potential malignancy (as mentioned in the context).

SCAI RAG Example from USMLE question in the Manuscript

Behavioral therapy treats Schizophrenic disorders. Behavioral therapy treats Binging. Behavioral therapy treats Tobacco dependence syndrome. Behavioral therapy treats Childhood asthma. Behavioral therapy prevents Chronic pain. Psychotherapy treats Moderate depression. Psychotherapy treats Chronic intractable pain. Urinalysis diagnoses Drug abuse. Behavioral therapy treats Mental disorder. Urinalysis diagnoses Membranous glomerulonephritis. Behavioral therapy prevents Suicide attempt. Psychotherapy treats Mood disorder. Urinalysis diagnoses Chlamydial infection. Psychotherapy treats Neurosis. Ankle exercises in water has as a direct procedure site Ankle region structure . Urinalysis diagnoses Chronic renal failure syndrome. Urinalysis diagnoses Asymptomatic bacteriuria. Behavioral therapy treats Nocturnal enuresis. Psychotherapy treats Psychological symptom. Behavioral therapy treats Urinary symptoms. Behavioral therapy treats Infection caused by Mycobacterium tuberculosis. Behavioral therapy prevents Migraine. Behavioral therapy treats Challenging behavior. Urinalysis diagnoses Torsion of spermatic cord. Psychotherapy treats Somatoform pain disorder. Psychotherapy treats Sequela. Uses oral contraception prevents Endometrial carcinoma. Psychotherapy treats Depressed mood. Urine culture diagnoses Chronic prostatitis. Psychotherapy treats Idiopathic torsion dystonia. Behavioral therapy treats Bipolar disorder. Behavioral therapy treats Heart failure. Psychotherapy treats Atopic dermatitis. Psychotherapy treats Panic disorder. Behavioral therapy treats Misuses drugs. Urine culture diagnoses Infection caused by Mycobacterium tuberculosis. Psychotherapy treats Delusions. Psychotherapy treats Seizure. Psychotherapy treats Body dysmorphic disorder. Psychotherapy treats Psychiatric symptom. Psychotherapy treats Heart failure. Behavioral therapy treats Dependent drug abuse. Behavioral therapy treats Atopic dermatitis. Behavioral therapy treats Self-injurious behavior. Urinalysis diagnoses Pyelonephritis. Behavioral therapy treats Major depressive disorder. Discussion about a problem is a focus of Problem. Uses oral contraception causes Lupus erythematosus. Behavioral therapy treats Anorexia nervosa. Psychotherapy treats Chronic pain. Behavioral therapy treats Migraine. Psychotherapy treats Co-morbid conditions. Urinalysis diagnoses Disorder of the urinary system. Uses oral contraception causes Amenorrhea. Behavioral therapy prevents Drug abuse. Psychotherapy treats Seasonal affective disorder. Behavioral therapy treats Headache. Behavioral therapy treats Dysgraphia. Behavioral therapy treats Blepharospasm. Behavioral therapy treats Eating disorder. Psychotherapy treats Symptoms of depression. Behavioral therapy treats Cerebral palsy. Urinalysis diagnoses Fever. Psychotherapy treats Dementia. Behavioral therapy

© 2025 Elkin PL et al. *JAMA Network Open*.

treats Chronic constipation. Behavioral therapy treats Chronic cough. Step ups treats Gastroesophageal reflux disease. Urinalysis diagnoses Abuse of laxatives. Behavioral therapy prevents Eating disorder. Uses oral contraception prevents Female pelvic inflammatory disease. Psychotherapy treats Migraine. Psychotherapy treats Obsessional thoughts. Psychotherapy treats Cocaine dependence. Urinalysis diagnoses Malignant epithelial neoplasm. Psychotherapy treats Abnormal sexual function. Psychotherapy treats School phobia. Urine culture diagnoses Urinary tract infectious disease. Determination of existing sensory impairments is a focus of Sensory nervous system finding. Urine culture diagnoses Recurrent urinary tract infection. Urinalysis diagnoses Urolithiasis. Psychotherapy prevents Suicide. Behavioral therapy treats Adipsia. Behavioral therapy prevents Insomnia. Behavioral therapy treats Infantile autism. Psychotherapy treats Dysthymia. Uses oral contraception causes Thrombosis of cerebral veins. Psychotherapy treats Attention deficit hyperactivity disorder, predominantly hyperactive impulsive type. Psychotherapy treats Recurrent major depressive episodes. Psychotherapy treats Ulcerative colitis. Psychotherapy treats Somatoform disorder. Psychotherapy treats Mania. Behavioral therapy treats Delusions. Psychotherapy treats Drug abuse. Psychotherapy treats Emotional problems. Psychotherapy treats Primary insomnia. Behavioral therapy treats Psychosexual disorder. Psychotherapy treats Major depression, single episode. Psychotherapy treats Seizure disorder. Uses oral contraception predisposes one to Venous thrombosis. Psychotherapy treats Rheumatoid arthritis. Behavioral therapy treats Psychophysiologic disorder. Behavioral therapy treats Impaired glucose tolerance. Behavioral therapy treats Impulse control disorder. Urinalysis diagnoses Acute tubular necrosis. Urinalysis diagnoses Porphyria cutanea tarda. Behavioral therapy treats Infertile. Behavioral therapy treats Excessive belching. Psychotherapy treats Disability. Behavioral therapy prevents Acquired immune deficiency syndrome. Behavioral therapy treats Non-alcoholic fatty liver. Psychotherapy treats Hyperhidrosis. Incontinence predisposes one to Pyelonephritis. Urinalysis diagnoses Renal vascular disorder. Psychotherapy treats Mild depression. Behavioral therapy treats Feeding problem. Psychotherapy treats Narcissistic personality disorder. Psychotherapy treats Chronic schizophrenia. Uses oral contraception prevents Malignant neoplastic disease. Psychotherapy treats Borderline personality disorder. Psychotherapy treats Raynaud's disease. Urinalysis diagnoses Vasculitis. Psychotherapy treats Nightmares. Behavioral therapy treats Rheumatism. Behavioral therapy treats Feeling suicidal. Behavioral therapy treats Seizure. Behavioral therapy treats Infantile colic. Psychotherapy treats Psychophysiologic disorder. Urinalysis diagnoses Glomerular disease. Urinalysis diagnoses Hypertensive disorder, systemic arterial. Urine culture diagnoses Cytomegalovirus infection. Behavioral therapy treats Psychological symptom. Urinalysis diagnoses Cystitis. Urine culture diagnoses Systemic inflammatory response syndrome. Behavioral therapy prevents Suicide. Psychotherapy treats Endogenous depression. Behavior management treats Schizophrenic disorders. Psychotherapy treats Alzheimer's disease. Psychotherapy treats Chronic depression. Psychotherapy treats Vitiligo. Behavioral therapy treats Social phobia. Behavioral therapy treats Stress. Uses oral contraception causes Thrombosis of mesenteric vein. Behavioral therapy treats Developmental disorder. Psychotherapy treats Disorder of gastrointestinal tract. Behavioral therapy treats Parkinson's disease. Behavioral therapy prevents Hypertensive disorder, systemic arterial. Urine culture diagnoses Pyelonephritis. Behavioral therapy treats Smoker. Psychotherapy treats Posttraumatic stress disorder. Behavioral therapy prevents Pain. Behavioral therapy treats Chronic low back pain. Behavioral therapy treats Periodontitis. Behavioral therapy treats Adult attention deficit hyperactivity disorder. Psychotherapy treats Obsessive-compulsive disorder. Psychotherapy treats Gender dysphoria. Behavioral therapy treats Difficulty breathing. Step ups treats Ulcerative colitis. Behavioral therapy treats Osteoarthritis of

© 2025 Elkin PL et al. *JAMA Network Open*.

knee. Behavioral therapy treats Neurosis. Urinalysis diagnoses Glomerulonephritis. Uses oral contraception prevents Disorder of breast. Behavioral therapy treats Insomnia. Behavioral therapy treats Dementia. Behavioral therapy prevents Pica. Behavioral therapy treats Mental handicap. Urinalysis, bacteriuria screen is a focus of Disorder of the genitourinary system. Psychotherapy treats Heart disease. Behavioral therapy treats Backache. Urinalysis diagnoses Hypercalciuria. Urinalysis diagnoses Urinary tract infectious disease. Behavioral therapy treats Coronary arteriosclerosis. Uses oral contraception prevents Functional cyst of ovary. Psychotherapy treats Mental disorder. Psychotherapy treats Pervasive developmental disorder. Behavioral therapy treats Obesity. Psychotherapy treats Personality disorder. Behavioral therapy treats Neurodevelopmental disorder. Behavioral therapy treats Drug abuse. Behavioral therapy treats Methamphetamine abuse. Urinalysis diagnoses Frank hematuria. Psychotherapy treats Hypertensive disorder, systemic arterial. Psychotherapy treats Delusional disorder. Behavioral therapy treats Postpartum depression. Psychotherapy treats Chronic insomnia. Psychotherapy prevents Major depressive disorder. Urine culture diagnoses Acute pyelonephritis. Behavioral therapy treats Panic. Urinalysis diagnoses Normal renal function. Psychotherapy treats Postpartum depression. Urinalysis diagnoses Acute appendicitis. Urinalysis diagnoses Malignant neoplasm of urinary bladder. Urinalysis diagnoses Malignant tumor of kidney. Incontinence causes Peripheral venous insufficiency. Psychotherapy treats Asthma. Timed up and go mobility test diagnoses Falls. Psychotherapy treats Eating disorder. Management of compliance with medication regimen is a focus of Encouragement of compliance with medication regimen . Behavioral therapy prevents Habit tic. Behavioral therapy treats Malignant neoplasm of breast. Assisting with activity of daily living is a focus of Finding of activity of daily living. Renewal of urethral prosthesis for incontinence is a focus of Incontinence. Behavioral therapy prevents Urinary incontinence. Psychotherapy treats Cannabis dependence. Behavioral therapy treats Symptoms of depression. Behavioral therapy treats Human immunodeficiency virus detected. Psychotherapy prevents Psychotic disorder. Psychotherapy treats Recurrent depression. Urine culture diagnoses Bacterial urinary infection. Behavioral therapy treats Hallucinations. Behavioral therapy treats Focal dystonia. Urinalysis diagnoses Infection caused by Helicobacter pylori. Psychotherapy treats Mental health problem. Psychotherapy treats Depersonalization disorder. Psychotherapy treats Schizophrenic disorders. Step ups treats Acute necrotizing pancreatitis. Psychotherapy prevents Borderline personality disorder. Psychotherapy treats Major depressive disorder. Psychotherapy treats Myofascial pain syndrome. Step ups treats Asthma. Uses oral contraception predisposes one to Myocardial infarction. Behavioral therapy treats Simple phobia. Urinalysis diagnoses Nephritic syndrome. Behavioral therapy treats Urinary incontinence. Psychotherapy treats Anorexia nervosa. Behavioral therapy treats Disruptive behavior. Behavioral therapy prevents Self-injurious behavior. Behavioral therapy treats Low back pain. Behavioral therapy prevents Lack of energy. Psychotherapy treats Essential hypertension. Behavioral therapy treats Disturbance in sleep behavior. Urinalysis diagnoses Tay-Sachs disease. Behavioral therapy prevents Severe obesity. Uses combined oral contraception predisposes one to Hypertensive disorder, systemic arterial. Behavioral therapy treats Cannabis dependence. Behavioral therapy treats Knee pain. Urinalysis diagnoses Glycosuria. Uses oral contraception causes Cerebral venous sinus thrombosis. Psychotherapy treats Histrionic personality disorder. Behavioral therapy treats Cocaine dependence. Psychotherapy treats Cluster B personality disorder. Behavioral therapy treats Essential hypertension. Psychotherapy treats Severe depression. Urine culture diagnoses Tuberculosis of genitourinary system. Behavioral therapy treats Temporomandibular joint disorder. Behavioral therapy treats Primary progressive apraxia of speech. Behavioral therapy treats Acute stress disorder.

© 2025 Elkin PL et al. *JAMA Network Open*.

Behavioral therapy treats Elective mutism. Psychotherapy treats Acne vulgaris. Pediatric activity of daily living needs assessment is a focus of Activity of daily living procedures and interventions. Urinalysis diagnoses Abnormal renal function. Behavioral therapy prevents Human immunodeficiency virus infection. Psychotherapy treats Functional disorder of gastrointestinal tract. Behavioral therapy treats Tic disorder. Urine culture diagnoses Chronic renal failure syndrome. Determination of existing sensory impairments has as a direct procedure site Structure of nervous system . Psychotherapy prevents Suicide attempt. Psychotherapy prevents Postpartum depression. Behavioral therapy treats Abnormal sexual function. Behavioral therapy treats Ischemic heart disease. Urinalysis diagnoses Albuminuria. Psychotherapy treats Masked depression. Psychotherapy treats Traumatic brain injury. Urinalysis diagnoses Pernicious anemia. Psychotherapy treats Adult attention deficit hyperactivity disorder. Behavioral therapy prevents Suicide attempt by inadequate means. Behavioral therapy treats Severe obesity. Psychotherapy treats Depressive disorder. Behavioral therapy treats Panic disorder with agoraphobia. Behavioral therapy treats Tension-type headache. Behavioral therapy treats Chronic insomnia. Urinalysis diagnoses Infection caused by Mycobacterium tuberculosis. Behavioral therapy treats Psychologic conversion disorder. Uses oral contraception causes Pulmonary embolism. Psychotherapy treats Disorder of skin. Behavioral therapy treats Primary insomnia. Behavioral therapy treats Postconcussion syndrome. Psychotherapy treats Premature ejaculation. Behavioral therapy treats Irritable bowel syndrome. Behavioral therapy treats Vaginal wall prolapse. Behavioral therapy treats Obstructive sleep apnea syndrome. Behavioral therapy treats Pain. Behavioral therapy treats Nightmares. Behavioral therapy treats Chronic fatigue syndrome. Behavior management treats Dementia. Behavioral therapy treats Sleep disorder. Psychotherapy prevents Symptoms of depression. Urinalysis diagnoses Renal insufficiency. Behavioral therapy treats Arthritis. Behavioral therapy treats Hypochondriasis. Psychotherapy treats Common duodenal ulcer. Escorting subject to toilet is a focus of Finding of activity of daily living. Behavioral therapy treats Intermittent explosive disorder. Urinalysis diagnoses Hemoglobinuria. Behavior management treats Migraine. Urinalysis diagnoses Hyponatremia. Psychotherapy treats Feeling suicidal. Behavioral therapy treats Stuttering. Behavioral therapy treats Menopausal symptom. Behavioral therapy treats Inflammatory bowel disease. Behavioral therapy treats Pedophilia. Behavioral therapy treats Anxiety disorder. Behavioral therapy treats Incontinence of feces. Behavioral therapy treats Overweight. Behavioral therapy treats Juvenile myoclonic epilepsy. Behavioral therapy treats Hyperactive behavior. Behavioral therapy prevents Malignant neoplasm of skin. Behavioral therapy treats Borderline personality disorder. Psychotherapy treats Suicidal. Psychotherapy treats Multiple personality disorder. Urinalysis diagnoses Crystalluria. Psychotherapy treats Pruritic disorder. Behavioral therapy treats Mental health problem. Behavioral therapy treats Malignant neoplasm of skin. Behavioral therapy prevents Obesity. Behavioral therapy treats Impaired cognition. Psychotherapy treats Dependent drug abuse. Psychotherapy treats Compulsive gambling. Activity of daily living needs assessment is a focus of Activity of daily living procedures and interventions. Urinary symptoms predisposes one to Urinary tract infectious disease. Psychotherapy treats Cocaine abuse. Urinalysis diagnoses Acute infectious disease. Psychotherapy treats Bipolar affective disorder, current episode depression. Psychotherapy treats Psychogenic voice disorder. Behavioral therapy treats Endogenous depression. Psychotherapy prevents Posttraumatic stress disorder. Behavioral therapy treats Hypertensive disorder, systemic arterial. Psychotherapy treats Binging. Behavior management treats Acquired brain injury. Uses combined oral contraception predisposes one to Venous thrombosis. Behavioral therapy prevents Symptoms of depression. Behavioral therapy prevents Sexually transmitted infectious disease. Psychotherapy treats Opioid dependence. Behavioral therapy

© 2025 Elkin PL et al. *JAMA Network Open*.

treats Premenstrual symptom. Uses oral contraception causes Venous thrombosis. Behavioral therapy prevents Chronic post-traumatic stress disorder. Psychotherapy treats Tuberculosis of respiratory system. Behavioral therapy treats Sexually transmitted infectious disease. Psychotherapy treats Psoriasis. Behavioral therapy prevents Nocturia. Behavioral therapy treats Body dysmorphic disorder. Urinalysis diagnoses Proteinuria. Uses oral contraception predisposes one to Thrombosis of cerebral veins. Behavioral therapy treats Generalized multiple sclerosis. Uses oral contraception predisposes one to Coronary arteriosclerosis. Behavioral therapy treats Problem drinker. Behavioral therapy treats Spasmodic torticollis. Behavioral therapy prevents Posttraumatic stress disorder. Urine culture diagnoses Bacteriuria. Psychotherapy treats Cluster C personality disorder. Behavioral therapy treats Hoarding. Behavioral therapy treats Persistent insomnia. Behavioral therapy treats Addiction. Psychotherapy treats Generalized anxiety disorder. Psychotherapy treats Mild major depression. Behavioral therapy prevents Aggressive behavior. Psychotherapy treats Headache. Psychotherapy treats Neurological symptom. Behavioral therapy treats Substance abuse. Behavioral therapy treats Adjustment disorder. Behavioral therapy treats Obsessive-compulsive disorder. Behavioral therapy treats Obsessional neurosis. Behavioral therapy treats Traumatic brain injury. Psychotherapy treats Persistent alcohol abuse. Behavioral therapy treats Pervasive developmental disorder. Urinalysis diagnoses Nephrotic range proteinuria. Urine culture diagnoses Proteus infection. Psychotherapy treats Bipolar disorder. Psychotherapy treats Stress. Psychotherapy treats Inflammatory bowel disease. Psychotherapy treats Bipolar II disorder. Psychotherapy treats Dizziness. Behavioral therapy treats Posttraumatic stress disorder. Behavioral therapy treats Personality disorder. Behavioral therapy treats Psychotic disorder. Urinalysis diagnoses Bacteriuria. Urine culture diagnoses Asymptomatic bacteriuria. Behavioral therapy treats Oropharyngeal dysphagia. Psychotherapy treats Malignant hypertension. Urinalysis diagnoses Microscopic hematuria. Incontinence care is a focus of Incontinence. Behavioral therapy prevents Caries of infancy. Psychotherapy treats Nonulcer dyspepsia. Psychotherapy treats Diabetes mellitus. Urinalysis diagnoses Pancreatitis. Behavioral therapy treats Peripheral vascular disease. Psychotherapy prevents Pain. Psychotherapy treats Hypochondriasis. Behavioral therapy treats Compulsive gambling. Psychotherapy treats Conduct disorder. Psychotherapy treats Peptic ulcer. Psychotherapy prevents Depressive disorder. Psychotherapy treats Self-injurious behavior. Behavioral therapy treats Rheumatoid arthritis. Urinalysis diagnoses Renal tubular disorder. Incontinence predisposes one to Pressure ulcer. Psychotherapy treats Pain. Behavioral therapy treats Cough. Urine culture diagnoses Fever. Behavioral therapy treats Chronic back pain. Behavioral therapy treats Alcohol abuse. Behavioral therapy treats Depressive disorder. Psychotherapy treats Insomnia. Behavioral therapy treats Raynaud's disease. Behavioral therapy treats Premature ejaculation. Behavioral therapy treats Fragile X syndrome. Psychotherapy treats Chronic post-traumatic stress disorder. Psychotherapy treats Chronic fatigue syndrome. Behavioral therapy treats Constipation. Behavioral therapy treats Stereotypy habit disorder. Behavioral therapy prevents Low back pain. Uses oral contraception causes Hypertensive disorder, systemic arterial. Behavioral therapy treats Vomiting. Incontinence care treats Dementia. Behavioral therapy treats Functional encopresis. Psychotherapy treats Alcohol abuse. Urinalysis diagnoses Sterile pyuria. Behavioral therapy treats Hypoglycemic disorder. Ankle exercises has as a direct procedure site Ankle region structure . Behavioral therapy treats Auditory hallucinations. Step ups has as a direct procedure site Knee region structure . Behavioral therapy treats Compulsive behavior. Psychotherapy treats Tension-type headache. Behavioral therapy treats Headache disorder. Psychotherapy treats Generalized multiple sclerosis. Urinalysis diagnoses Acute intermittent porphyria. Urinalysis diagnoses Ectopic pregnancy. Uses oral contraception predisposes one to Cerebrovascular

© 2025 Elkin PL et al. *JAMA Network Open*.

accident. Behavioral therapy treats Generalized anxiety disorder. Urinalysis diagnoses Myoglobinuria. Urinalysis diagnoses Urinary tract obstruction. Psychotherapy treats Anxiety disorder. Psychotherapy treats Infertile. Behavioral therapy treats Microalbuminuria. Psychotherapy treats Disturbance in sleep behavior. Behavioral therapy prevents Headache. Behavioral therapy treats Persistent alcohol abuse. Behavioral therapy treats Obsessional thoughts. Behavioral therapy treats Asthma. Urinalysis diagnoses Malignant tumor of prostate. Psychotherapy treats Irritable bowel syndrome. Behavioral therapy prevents Tic disorder. Abnormal vital signs predisposes one to Pneumonia. Urinalysis diagnoses Ketoacidosis due to diabetes mellitus. Urine culture diagnoses Bacterial pneumonia. Psychotherapy treats Psychotic disorder. Behavioral therapy prevents Overweight. Uses oral contraception causes Deep venous thrombosis. Behavior management treats Obesity. Behavioral therapy treats Panic disorder. Psychotherapy treats Neurodermatitis. Behavioral therapy prevents Disturbance in sleep behavior. Behavioral therapy treats Posttraumatic headache. Urinalysis diagnoses Kidney disease. Urinalysis diagnoses Primary bacterial peritonitis. Psychotherapy treats Obesity. Psychotherapy treats Mental retardation. Behavioral therapy treats Abdominal pain. Behavioral therapy treats Chronic pain. Uses oral contraception predisposes one to Deep venous thrombosis. Psychotherapy treats Disorder of endocrine system. Urinalysis diagnoses Dihydropyrimidinase deficiency.

High-Definition NLP Process

The workflow for the HD-NLP software is most simply represented as an input processor that knows many input formats including many / most types of medical records (which can be modularly expanded for new use-cases). The input is parsed by an ontological encoder into a full semantic parse in memory including part of speak tagging, then assignment of ontology terms from as many ontologies as you like, then truth tagging as a seven valued logic (True, almost certainly true, more true than false, equaly likely to be true or false, more likely false than true, almost certainly false, false) with strong inter-rater reliability, then date and time tagging and identification of co-reference (eFigure 1).

© 2025 Elkin PL et al. *JAMA Network Open*.

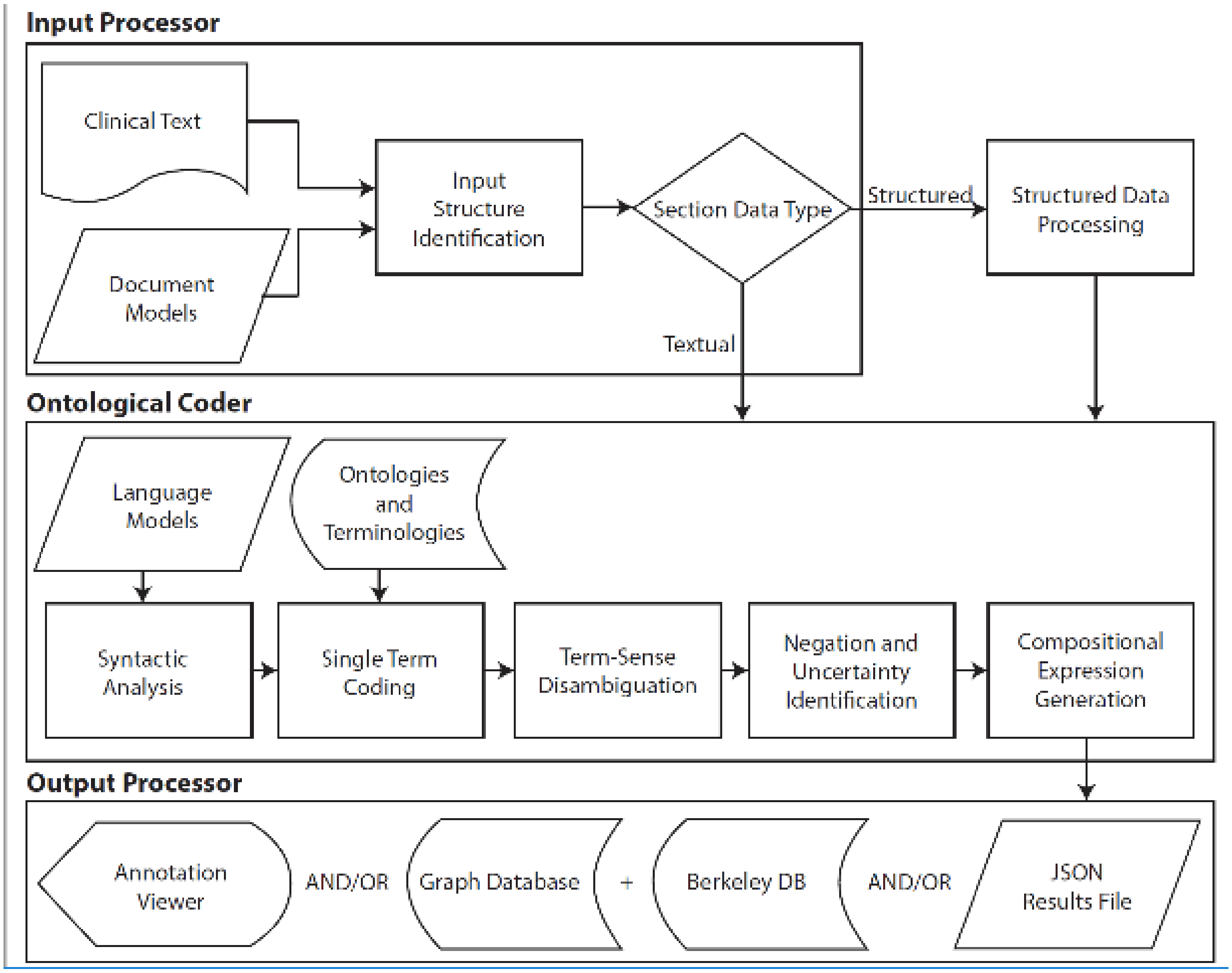

eFigure 1: High-Level HD-NLP Architecture

The HD-NLP is architected using the Unstructured Information Management Architecture (UIMA). There are broadly 4 different types of components that go into a given HD-NLP pipeline:

Low level linguistics: Including sentence identification and part-of speech tagging.

Terminologies: This is matching parts of the text in a document up with terms in an ontology such as SNOMED, Loinc, RxNorm, etc. including synonyms in the terms, variations of word order and word sense disambiguation. This uses a computational linguistics background and for word sense disambiguation we use a machine learning approach.

Text Section Grammars: These are processors that select larger areas of text than is contained in the terminologies and creates relations within them, using a hierarchical grammar formalism. These grammars can be used to identify things like what the header of a text section is, or syntactically defined entities such as quantities and dates. More importantly these grammars are used to identify relations between entities such as associating a quantity with a drug. These grammars are used to identify whether an entity is being negated.

© 2025 Elkin PL et al. *JAMA Network Open*.

Post Processing: These are UIMA analysis engines that operate on the previously found entities, such as eliminating ambiguous terms or whether an identified entity is a subpart of a larger entity, e.g. “lung” is a part of “lung cancer” and we thus may not need to process it separately.

Depending on the situation and challenges these processes can be run sequentially or sometimes in parallel. For example, the machine learning models for disambiguating whether the SNOMED code for “cancer tumor” verses “cancer the disease”, may be much slower than the process which identifies “cancer” as a SNOMED code initially. In this case, multiple process or machines running the disambiguation routine can be running simultaneously.

Architecture

When running the HD-NLP in a typical application there are typically two stages: the ingest stage and the query stage.

Ingest

During the ingest stage, data is taken in from a source and made searchable. Data is often coming from a variety of sources that may by inconsistent or incompatible. Some data will be structured data stored in a database; some may be text files in a directory.

Structured data with prespecified codes are directly indexed in the code index as SOLOR UUIDs and SNOMED CT, LOINC and RxNorm codes, which is typically stored in Elastic Search. We have found that Elastic Search provides much faster indexing than other available alternatives such as storage in a relational database, but other options are available including usage of NoSQL databases and triple stores and graph databases like Neo4J.

Depending on how the data is being ingested natural language data is often sent to a messaging queue for processing by the HD-NLP. Message queues that have been used include RabbitMQ and Amazon SQS. The use of a message queue helps enable scalability with regard to the HD-NLP process, allowing HD-NLP processes to be added (often in Docker Containers) with little configuration. The results of the HD-NLP process are then added to the Elastic Search index. Codes are indexed in the same way as codes from structured text but often relations between codes are indexed as well.

Search

HD-NLP processing is done on the search side as well. Natural language queries as well as medical ontology codes can be used to find indexed health records. A natural language query goes into the HD-NLP process to produce codes to be searched, including relations between codes and entities and whether the code is negated for boolean search. A search term such as “heart disease without diabetes” will return the set of documents that have a code for heart disease but also are explicitly negated for diabetes (eFigure 2).

© 2025 Elkin PL et al. *JAMA Network Open*.

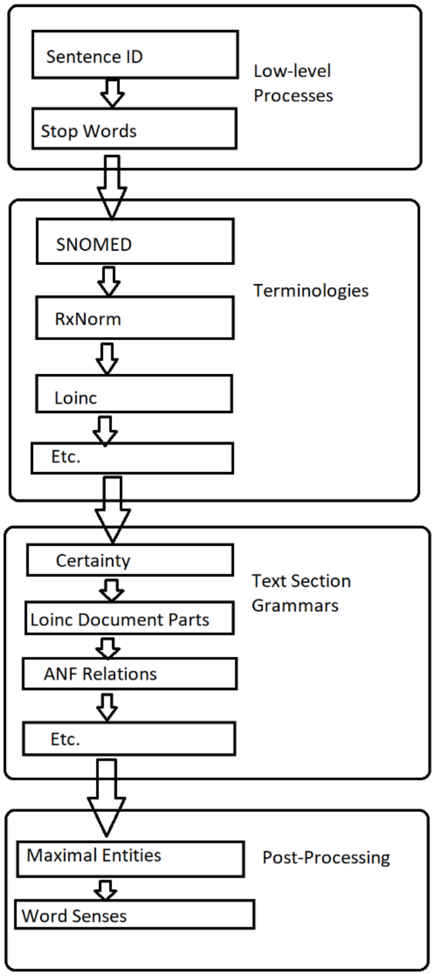

eFigure 2: Logic flow for the HD-NLP pipeline

© 2025 Elkin PL et al. *JAMA Network Open*.

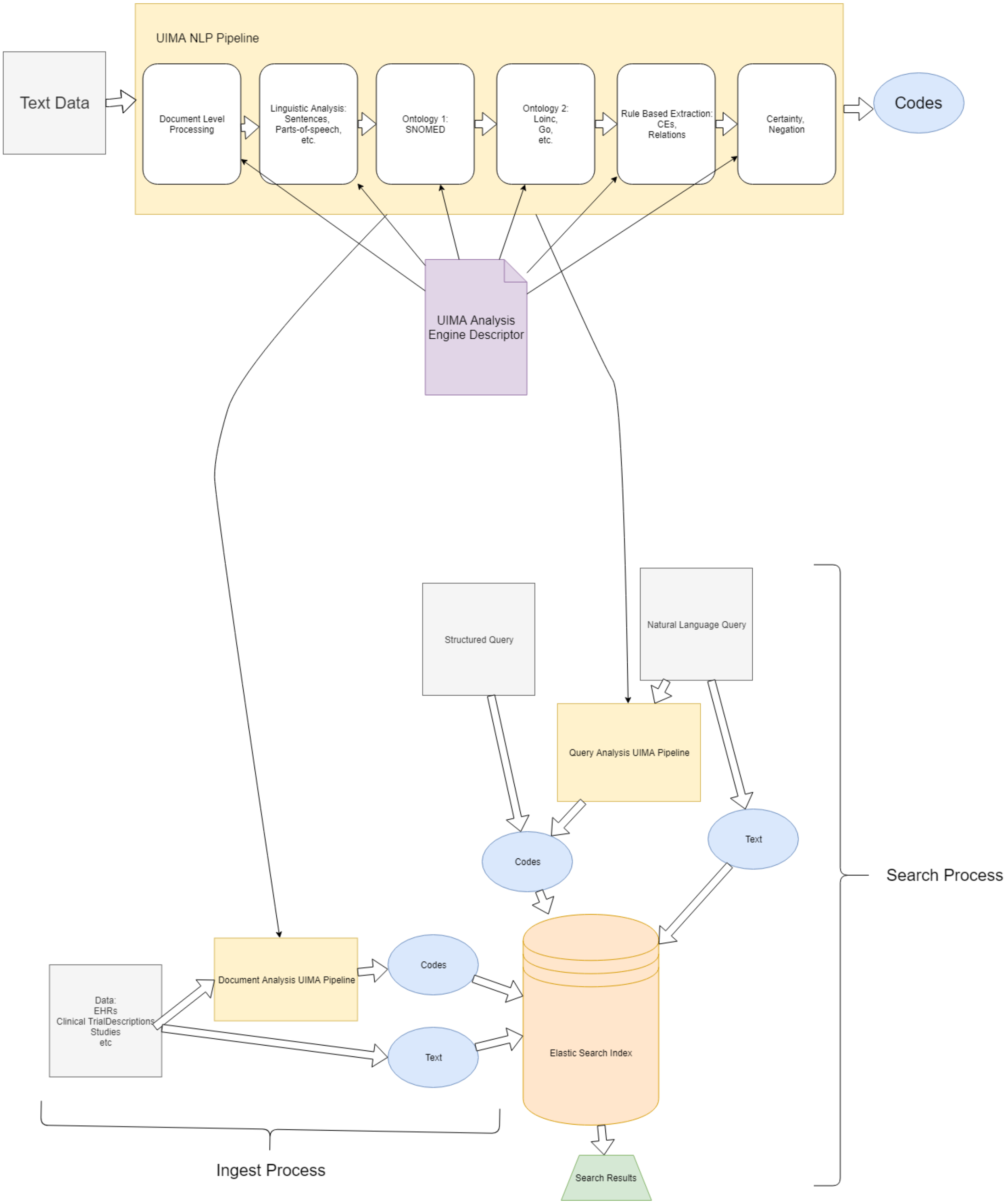

eFigure 3: This logic flow includes both the HD-NLP and also the data ingestion using HD-NLP and Structured data (e.g. OMOP) and the pathway for access to both structured and unstructured data in one query.

As a general natural language processor this problem is somewhat different than many others in that it usually involves finding bits of natural language text that correspond to a very large pre-

© 2025 Elkin PL et al. *JAMA Network Open*.

defined set of terms.   Because there are many thousands of concepts in SNOMED for example, it makes the techniques that were used by earlier information extraction systems to identify the names of people, places and organizations, for example, fairly useless.

For example, the NIST Automatic Content Extraction competitions (https://en.wikipedia.org/wiki/Automatic_content_extraction) that were once the gold standard for evaluating information extraction systems included only a few dozen concepts altogether. This meant that system developers were able to use human annotated training data that included all of the concepts in their ontology many times over in order to apply a machine learning algorithm to categorize text into those few concepts.

By contrast, even extremely large sets of medical documents may never include an example of all the concepts listed in SNOMED and hand annotating such a data set to match all of those concepts would likely be impossible.

Consequently, HD-NLP relies on taking a different path to the information extraction problem by looking at the descriptions of the ontological concepts that it is trying to extract and coming up with as many possible variations of text for each of those concepts that it can possibly account for. If SNOMED-CT includes the concept 25370001 - Hepatocellular Carcinoma, the possible variations of text that might trigger this concept might include, “Liver Cell Carcinoma”, “Liver Cell Cancer”, “Liver Cancer”, “cancer of the liver”, etc. Through both automatic means and human enhancement, BMI-HTP maintains a language model that includes all the possible synonyms that could occur (eFigure 3).

Additionally, HD-NLP is able to extract relations that exist between ontology concepts. For example if a medicine is extracted, the system should be able to relate that medicine to the dosage. If a tumor is described in the text and the text also specifies where in the body the tumor is located, the system should be able to provide that relation.   As with the synonymy set, relations are also extensible.

UIMA

The NLP pipeline which inputs text and outputs medical codes conforms to a standardized API called UIMA (https://www.oasis-open.org/committees/uima/charter.php) and is implemented using the most popular java library for this API, Apache UIMA (https://uima.apache.org/). Please see the documentation at these two sources, particularly the latter to see how to integrate library directly into an application using this API.   The rest of this section contains details on the configuration of the system from the perspective of a UIMA pipeline.

© 2025 Elkin PL et al. *JAMA Network Open*.

SCAI Interface (eFigure 4 below).

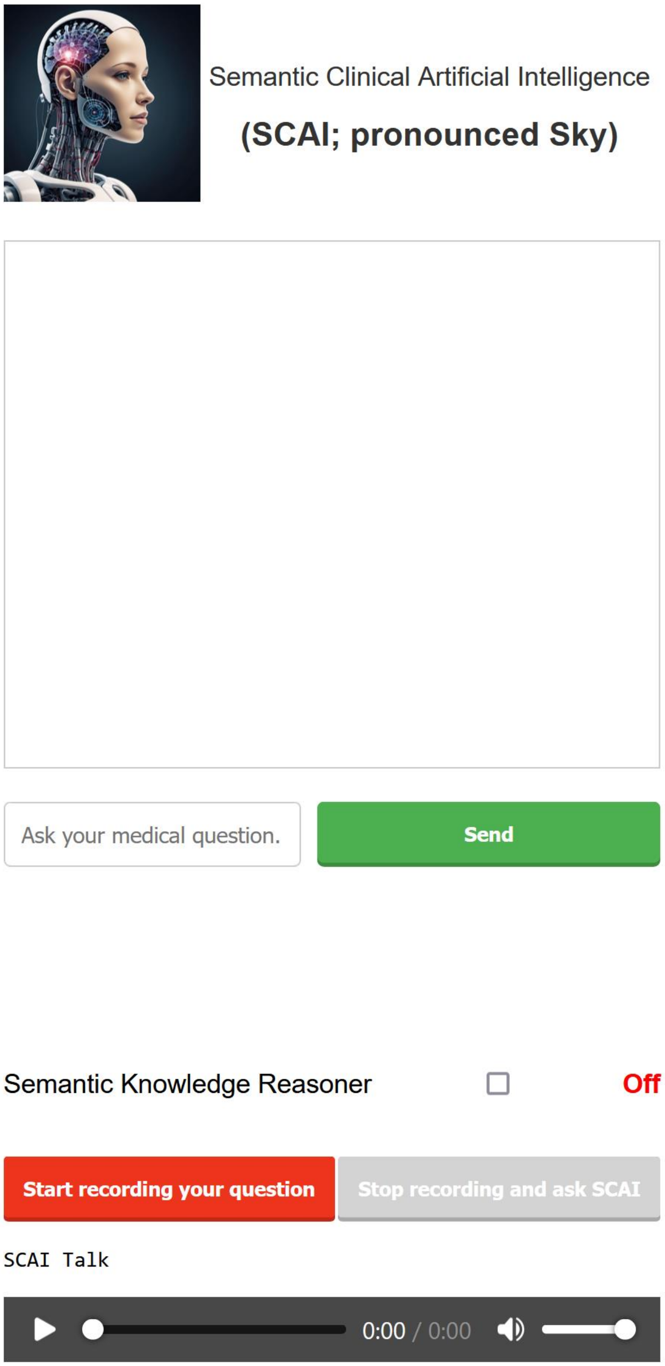

eFigure 4 – Semantic Clinical Artificial Intelligence Interface with text or voice recognition input and answers

© 2025 Elkin PL et al. *JAMA Network Open*.
