# Supplementary material for: Semantic Clinical Artificial Intelligence vs Native Large Language Model Performance on the USMLE
Source: JAMA Netw Open. 2025 Apr 22;8(4):e256359. doi: 10.1001/jamanetworkopen.2025.6359 (PMC12015668; doi:10.1001/jamanetworkopen.2025.6359)
Supplement: Supplement 2. — Data Sharing Statement [file jamanetwopen-e256359-s002.pdf]

Data Sharing Statement

Elkin. Semantic Clinical Artificial Intelligence vs Native Large Language Model Performance on the USMLE. *JAMA Netw Open*. Published April 22, 2025.
doi:10.1001/jamanetworkopen.2025.6359

Data
Data available: Yes
Data types: Data (not involving human participants)
How to access data: The USMLE questions and answers will be made available. The Llama model is open source.
When available: With publication

Supporting Documents
Document types: Statistical/analytic code
How to access documents: McNemar test
When available: With publication

Additional Information
Who can access the data: anyone requesting the data,
Types of analyses: for any purpose
Mechanisms of data availability: upon request and without support
